# Multi-method Fusion of Cross-Subject Emotion Recognition Based on High-Dimensional EEG Features

**DOI:** 10.3389/fncom.2019.00053

**Published:** 2019-08-20

**Authors:** Fu Yang, Xingcong Zhao, Wenge Jiang, Pengfei Gao, Guangyuan Liu

**Affiliations:** ^1^College of Electronic Information and Engineering, Southwest University, Chongqing, China; ^2^Chongqing Key Laboratory of Nonlinear Circuit and Intelligent Information Processing, Chongqing, China

**Keywords:** EEG, emotion recognition, cross-subject, multi-method fusion, high-dimensional features

## Abstract

Emotion recognition using electroencephalogram (EEG) signals has attracted significant research attention. However, it is difficult to improve the emotional recognition effect across subjects. In response to this difficulty, in this study, multiple features were extracted for the formation of high-dimensional features. Based on the high-dimensional features, an effective method for cross-subject emotion recognition was then developed, which integrated the significance test/sequential backward selection and the support vector machine (ST-SBSSVM). The effectiveness of the ST-SBSSVM was validated on a dataset for emotion analysis using physiological signals (DEAP) and the SJTU Emotion EEG Dataset (SEED). With respect to high-dimensional features, the ST-SBSSVM average improved the accuracy of cross-subject emotion recognition by 12.4% on the DEAP and 26.5% on the SEED when compared with common emotion recognition methods. The recognition accuracy obtained using ST-SBSSVM was as high as that obtained using sequential backward selection (SBS) on the DEAP dataset. However, on the SEED dataset, the recognition accuracy increased by ~6% using ST-SBSSVM from that using the SBS. Using the ST-SBSSVM, ~97% (DEAP) and 91% (SEED) of the program runtime was eliminated when compared with the SBS. Compared with recent similar works, the method developed in this study for emotion recognition across all subjects was found to be effective, and its accuracy was 72% (DEAP) and 89% (SEED).

## 1. Introduction

Emotion is essential to humans, as it contributes to the communication between people and plays a significant role in rational and intelligent behavior (Picard et al., 2001; Nie et al., 2011), which is critical to several aspects of daily life. Therefore, research on emotion recognition is necessary. It is difficult to define and classify emotion due to the complex nature and genesis of emotion (Ashforth and Humphrey, 1995; Horlings et al., 2008; Hwang et al., 2018). To classify and represent emotion, several models have been proposed. Moreover, there are two main models. The first assumes that all emotions can comprise primary emotions, similar to how all colors can comprise primary colors. Plutchik (1962) related eight basic emotions to evolutionarily valuable properties, and then reported the following primary emotions: anger, fear, sadness, disgust, surprise, curiosity, acceptance, and joy. Ekman (Power and Dalgleish, 1999; Horlings et al., 2008) reported other emotions as a basis set and found that these primary emotions, in addition to their expressions, are universal. The Ekman list of primary emotions is as follows: anger, fear, sadness, happiness, disgust, and surprise. The second main model is composed of multiple dimensions, and each emotion is on a multi-dimensional scale. Russell (1980) divided human emotions into two dimensions: arousal and valence. Arousal represents the strength of the emotion with respect to arousal and relaxation and valence represents positive and negative levels. Among several emotional models, the Russell model (Russell, 1980) is generally adopted, in which two dimensions are represented by a vertical arousal axis and horizontal valence axis (Choi and Kim, 2018). In both dimensions of emotion, the ability to measure valence levels is essential, as the valence level is a more critical dimension for distinguishing between positive emotions (e.g., excitement, happiness, contentment, or satisfaction) and negative emotions (e.g., fear, anger, frustration, mental stress, or depression; Hwang et al., 2018). It is necessary to effectively classify and identify positive and negative emotions. For example, the accurate identification of the mental stress (a negative emotion) or emotional state of construction workers can help reduce construction hazards and improve production efficiency (Chen and Lin, 2016; Jebelli et al., 2018a). The focus of this study was on improving the classification accuracy of positive and negative emotions. In daily human life, communication and decision-making are influenced by emotional behavior. For many years, the brain-computer interface (BCI) has been a critical topic with respect to biomedical engineering research, allowing for the use of brain waves to control equipment (Nijboer et al., 2009). To achieve accurate and smooth interactions, computers and robots should be able to analyze emotions (Pessoa and Adolphs, 2010; Zheng et al., 2019). Researchers in the fields of psychology, biology, and neuroscience have directed significant attention toward emotional research. Emotional research has a preliminary development trend in the field of computer science, such as task workload assessments and operator vigilance (Shi and Lu, 2013; Zheng and Lu, 2017b). The automatic emotion recognition system simplifies the computer interface and renders it more convenient, more efficient, and more user-friendly. Human emotion recognition can be studied using questionnaires (Mucci et al., 2015; Jebelli et al., 2019), facial images, gestures, speech signals and other physiological signals (Jerritta et al., 2011). However, the questionnaire method interfered with this study. In addition, it exhibited a significant deviation and yielded inconsistent results (Jebelli et al., 2019). There was an ambiguity with respect to emotion recognition from facial images, gestures, or speech signals, as real emotions can be mimicked. To overcome the ambiguity, an electroencephalogram (EEG) could be employed for emotion recognition, as it is more accurate and more objective than emotional evaluation based on facial image and gesture-based methods (Ahern and Schwartz, 1985). Therefore, EEG has attracted significant research attention. Moreover, EEG signals can be used to effectively identify different emotions (Sammler et al., 2007; Mathersul et al., 2008; Knyazev et al., 2010; Bajaj and Pachori, 2014). For effective medical care, it is essential to consider emotional states (Doukas and Maglogiannis, 2008; Petrantonakis and Hadjileontiadis, 2011). Due to the objectivity of physiological data and the ability to model learning principles from heterogeneous features to emotional classifiers, the use of machine learning methods for the analysis of EEG signals has attracted significant attention in the field of human emotion recognition. To improve the satisfaction and reliability of the people who interact and collaborate with machines and robots, a smart human-machine (HM) system that can accurately interpret human communication capabilities is required (Koelstra et al., 2011). Human intentions and commands mostly convey emotions in a linguistic or non-verbal manner; thus, the accurate response to human emotional behavior is critical to the realization of machine and computer adaptation (Zeng et al., 2008; Fanelli et al., 2010). At this stage, the majority of HM systems cannot accurately recognize emotional cues. Emotional classifiers were developed based on facial/sound expressions or physiological signals (Hanjalic and Xu, 2005; Kim and André, 2008). Emotion classifiers can provide temporal predictions of specific emotional states. Emotional recognition requires appropriate signal preprocessing techniques, feature extraction, and machine learning-based classifiers to carry out automatic classification. An EEG, which captures brain waves, can effectively distinguish between emotions. The EEG directly detects brain waves from the central nervous system activities (i.e., brain activities), whereas other responses (e.g., EDA, HR, and BVP) are based on peripheral nervous system activities (Zhai et al., 2005; Chanel et al., 2011; Hwang et al., 2018). In particular, central nervous system activities are related to several aspects of emotions (e.g., from displeasure to pleasure, and from relaxation to excitement); however, the peripheral nervous system activities are only associated with arousal and relaxation (Zhai et al., 2005; Chanel et al., 2011). Therefore, the EEG can provide more detailed information on emotional states than other methods (Takahashi et al., 2004; Lee and Hsieh, 2014; Liu and Sourina, 2014; Hou et al., 2015). Moreover, EEG-based emotion recognition has a greater potential with respect to research than facial and speech-based methods, given that internal nerve fluctuations cannot be deliberately masked or controlled. However, the improvement of the performance of cross-subject emotional recognition has been the focus of several studies, including this study. In previous studies, cross-subject emotion recognition was difficult to achieve when compared with intra-subject emotion recognition. In Kim (2007), the method of bimodal data fusion was investigated, and a linear discriminant analysis (LDA) was conducted to classify emotions. The best recognition accuracy across the three subjects was 55%, which was significantly lower than the 92% achieved using the intra-subject emotion recognition method (Kim, 2007). In Zhu et al. (2015), the authors employed differential entropy (DE) as features, and a linear dynamic system (LDS) was applied to carry out feature smoothing. The average cross-subject classification accuracy was 64.82%, which was significantly lower than the 90.97% of the intra-subject emotion recognition method (Zhu et al., 2015). In Zhuang et al. (2017), a method for feature extraction and emotion recognition based on empirical mode decomposition (EMD) was introduced. Using EMD, the EEG signals were automatically decomposed into intrinsic mode functions (IMFs). Based on the results, IMF1 demonstrated the best performance, which was 70.41% for valence (Zhuang et al., 2017). In Candra et al. (2015), an accuracy of 65% was achieved for valence and arousal using the wavelet entropy of signal segments with periods of 3–12 s. In Mert and Akan (2018), the advanced properties of EMD and its multivariate extension (MEMD) for emotion recognition were investigated. The multichannel IMFs extracted by MEMD were analyzed using various time- and frequency-domain parameters such as the power ratio, power spectral density, and entropy. Moreover, Hjorth parameters and correlation were employed as features of the valence and arousal scales of the participants. The proposed method yielded an accuracy of 72.87% for high/low valences (Mert and Akan, 2018). In Zheng and Lu (2017a), deep belief networks (DBNs) were trained using differential entropy features extracted from multichannel EEG data, and the average accuracy was 86.08%. In Yin et al. (2017), cross-subject EEG feature selection for emotion recognition was carried out using transfer recursive feature elimination. The classification accuracy was 78.75% in the valence dimension, which was higher than those reported in several studies that used the same database. However, from the calculation times of all the classifiers, it was found that the accuracy of the *t*-test/recursive feature extraction (T-RFE) increased at the expense of the training time. In Li et al. (2018), 18 linear and non-linear EEG features were extracted. In addition, the support vector machine (SVM) method and the leave-one-subject-out verification strategy were used to evaluate the recognition performance. With the automatic feature selection method, the recognition accuracy rate using the dataset for emotion analysis using physiological signals (DEAP) was a maximum of 59.06%, and the recognition accuracy using the SEED dataset was a maximum of 83.33% (Li et al., 2018). In Gupta et al. (2018), the aim of the study was to comprehensively investigate the channel specificity of EEG signals and provide an effective emotion recognition method based on the flexible analytic wavelet transform (FAWT). The average classification accuracy obtained using this method was 90.48% for positive/neutral/negative (SEED) emotion classification, and 79.99% for high valence (HV)/low valence (LV) emotion classification using EEG signals (Gupta et al., 2018). In Li et al. (2019), the accuracy of multisource supervised STM (MS-S-STM) for emotion recognition accuracy was 88.92%, and the multisource semi-supervised selective transfer machine (STM) (MS-semi-STM) experimental data was used in a transmissive manner, with a maximum accuracy of 91.31%. The methods of emotion recognition across subjects, as employed in the previous studies, require improvements. A method for improving the accuracy of emotion classification is therefore necessary, which requires only a small computational load when applied to the analysis of high-dimensional features. In this study, multiple types of features were extracted. In addition, a two-category emotion recognition method across subjects is proposed. In particular, 10 types of linear and non-linear EEG features were first extracted, and then combined into high-dimensional features. With respect to high-dimensional features, a method for improving the emotion recognition performance across subjects based on high-dimensional features was proposed. Moreover, the significant test/sequential backward selection/support vector machine (ST-SBSSVM) fusion method was proposed and then used to identify and classify the high-dimensional EEG features of the cross-subject emotions.

## 2. Materials and Methods

**Figure 1:**
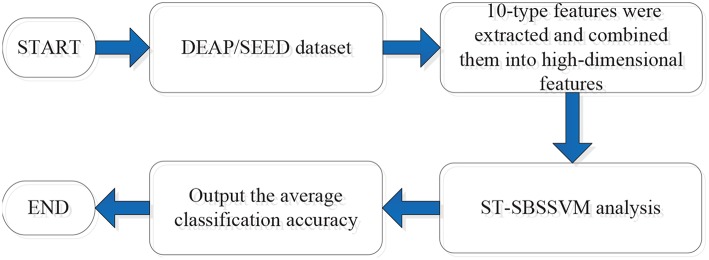
Analysis process.

Figure 1 presents the analysis process in this study. First, 10 types of high-dimensional features were extracted from both the DEAP and SEED. The features were then combined into high-dimensional features, as follows:

(1) $$\begin{array}{l} DEAP,1280\left(trials,rows\right)\times 320\left(features,cols\right) \end{array}$$

(2) $$\begin{array}{l} \;SEED,450\left(trials,rows\right)\times 620\left(features,cols\right) \end{array}$$

For further details, refer to section 2.2. Furthermore, the proposed method (ST-SBSSVM) was used to analyze the high-dimensional features and output the classification and recognition accuracy of positive and negative emotions.

### 2.1. DEAP Dataset and SEED Dataset

Two publicly accessible datasets were employed for the analysis, namely, the DEAP and SEED. The DEAP dataset (Koelstra et al., 2011) consisted of 32 subjects. Each subject was exposed to 40 1-min long music videos as emotional stimuli while their physiological signals were recorded. The resulting dataset includes 32 channels of EEG signals, four-channel electrooculography (EOG), four-channel electromyography (EMG), respiration, plethysmography, galvanic skin response and body temperature. Each subject underwent 40 EEG trials, each of which corresponded to an emotion triggered by a music video. After watching each video, the participants were asked to score their real emotions on a five-level scale: (1) valence (related to the level of pleasure), (2) arousal (related to the level of excitement), (3) dominance (related to control), (4) like (related to preference), and (5) familiarity (related to the awareness of stimuli). The score ranged from 1 (weakest) to 9 (strongest), with the exception of familiarity, which ranged from 1 to 5. The EEG signal was recorded using Biosemi ActiveTwo devices at a sampling frequency of 512 Hz and down-sampling frequency of 128 Hz. The data structure of DEAP is shown in Table 1. The SEED (Zheng and Lu, 2017a) consisted of 15 subjects. Movie clips were selected to induce (1) positive emotions, (2) neutral emotions, and (3) negative emotions; each of which were distributed over five segments of each movie. All subjects underwent three EEG recordings, with two consecutive recordings conducted at a two-week interval. At each stage, each subject was exposed to 15 movie clips, each of which was ~4 min long, to induce specific emotions. The same 15-segment movie clip was used in all three recording sessions. The resulting data contained 15 EEG trials. Each subject underwent 15 trials with 5 trials per emotion. The EEG signals were recorded using a 62-channel NeuroScan electric source imaging (ESI) device at a sampling rate of 1,000 Hz and down-sampling rate of 200 Hz. The data structure of SEED is shown in Table 2 (the duration of the SEED videos varied: each video was about 4 min = 240 s; thus, the data were about 200*Hz* × 240*s* = 48, 000). In this study, only experiments with positive emotions and negative emotions were carried out to evaluate the ability of the proposed method to distinguish between these two emotions. For consistency with the DEAP, 1 min of data extracted at the middle of each trial was employed using the SEED.

**Table 1:** Structure of the DEAP dataset.

**Array name**	**Array shape**	**Array contents**
Data	40 × 40 × 8, 064	(*Videos*/*trials*) × *channels* × *data* (128*Hz* × 63*s*)
Labels	40 × 4	(*videos*/*trials*) × *labels* (*Valence, arousal, dominance, liking*)

**Table 2:** Structure of the SEED dataset.

**Array name**	**Array shape**	**Array contents**
Data	3 × 15 × 62 × 48, 000	(*Experiments*) × (*Videos*/*trials*) × *channels*
		× *data* (200*Hz* × 240*s*)
Labels	3 × 15 × 3	(*Experiments*) × (*videos*/*trials*) × *labels*
		(*positive, neutral, negative*)

### 2.2. Data Processing

#### 2.2.1. Data Preprocessing

The EEG signal considered in this study was a neurophysiological signal with a high dimensionality, redundancy, and noise. After the EEG data were collected, the original data were pre-processed, i.e., the removal of EOG, EMG artifacts, and down-sampling; to reduce the computational overhead of feature extraction. For the DEAP, the default pre-processing technique was as follows: (1) the data was down-sampled to 128 Hz; (2) the EOG artifacts were removed, as achieved in Koelstra et al. (2011); (3) a bandpass filter with a throughput frequency range of 4.0–45.0 Hz was applied; (4) the data were averaged to the common reference; and (5) the data were segmented into 60-s trials and a 3-second pre-trial baseline. For the SEED, the default preprocessing technique was applied as follows: (1) the data was down-sampled to 200 Hz; (2) a bandpass filter with a throughput frequency range of 0–75 Hz was applied; and (3) the EEG segments corresponding to the duration of each movie were extracted. Prior to the extraction of the power spectral density (PSD) features, four rhythms were extracted, namely, theta (3–7 Hz), alpha (8–13 Hz), beta (14–29 Hz), and gamma (30–47 Hz) (Koelstra et al., 2011). Other features were extracted on the data preprocessed by the dataset.

#### 2.2.2. Label Processing

For label processing using the DEAP, the subjects were divided into two categories according to the corresponding scores of the subjects with respect to valence. A score higher than 5 was set as 1, which represented positive emotions; and a score below 5 was set as 1, which represented negative emotions. In the SEED, the trials were divided into positive emotions, neutral emotions, and negative emotions. However, for consistency with the DEAP, only positive and negative emotion samples were investigated using the SEED. Moreover, binary classification tasks were employed to carry out emotional recognition across the subjects.

### 2.3. Feature Extraction

**Figure 2:**
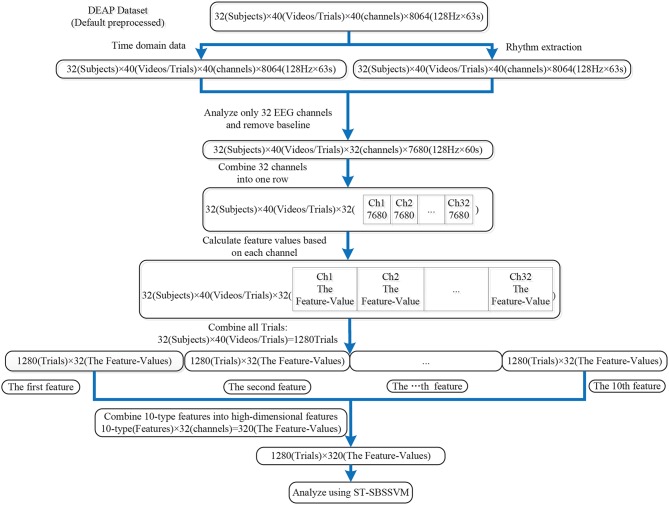
DEAP data analysis and feature extraction process.

**Figure 3:**
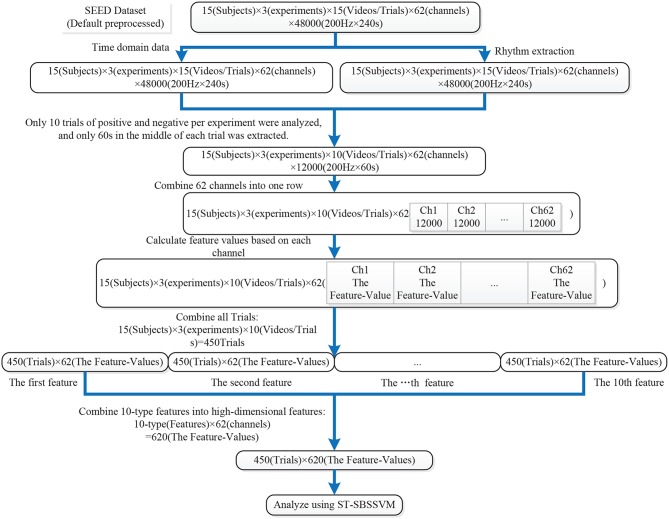
SEED data analysis and feature extraction process.

Ten types of linear and non-linear features were extracted, as shown in Table 3. Several features [Hjorth activity, Hjorth mobility, Hjorth complexity, standard deviation, sample entropy (SampEn), and wavelet entropy (WE)] were directly extracted from the dataset pre-processed EEG signals. The extraction processes of the remaining features (the four PSD frequency domain features) were divided into two steps. First, four types of rhythms were extracted from the EEG signals pre-processed using the dataset, and the PSD features were then extracted from the four rhythms. The detailed analysis of the data and feature extraction is shown in Figures 2, 3.

**Table 3:** Ten-type EEG features.

**Feature type**	**Extracted features**		
The linear features	1. Hjorth activity	2. Hjorth mobility	3. Hjorth complexity
	4. The standard deviation	5. PSD-Alpha	6. PSD-Beta
	7. PSD-Gamma	8. PSD-Theta	
The non-linear features	9. Sample entropy	10. Wavelet entropy	

#### 2.3.1. The Linear Feature

Hjorth parameters were indicators of statistical properties used in signal processing in the time domain, as introduced by Hjorth (1970). The parameters are as follows: activity, mobility, and complexity. They were commonly used in the analysis of electroencephalography signals for feature extraction. The parameters are normalized slope descriptors (NSDs) used in EEGs. The standard deviation feature was the standard value of the EEG time-series signal. The four PSD Features were extracted as follows: PSD-alpha was extracted from the alpha rhythm, PSD-beta was extracted from the beta rhythm, PSD-gamma was extracted from the gamma rhythm, and PSD-theta was extracted from the theta rhythm. The power spectrum *S*_*xx*_(*f*) of a time series *x*(*t*) describes the power distribution with respect to the frequency components that compose that signal (Fanelli et al., 2010). According to Fourier analysis, any physical signal can be decomposed into several discrete frequencies, or a spectrum of frequencies over a continuous range. The statistical average of a certain signal or signal type (including noise), as analyzed with respect to its frequency content, is referred to as its spectrum. When the energy of the signal is concentrated around a finite time interval, especially if its total energy is finite, the energy spectral density can be computed. Moreover, the PSD (power spectrum) is more commonly used, which applies to signals existing over a sufficiently large time period (especially in relation to the duration of a measurement) that can be considered as an infinite time interval. The PSD refers to the spectral energy distribution per unit time, given that the total energy of such a signal over an infinite time interval would generally be infinite. The summation or integration of the spectral components yield the total power (for a physical process) or variance (in a statistical process), which correspond to the values that are obtained by integrating *x*^2^(*t*) over the time domain, as dictated by Parseval's theorem (Snowball, 2005). For continuous signals over a quasi-infinite time interval, such as stationary processes, the PSD) should be defined, which describes the power distribution of a signal or time-series with respect to frequency.

#### 2.3.2. The Non-linear Feature

The SampEn is a modification of the approximate entropy (ApEn), and it is used for assessing the complexity of physiological time-series signals in addition to the diagnosis of diseased states (Richman and Moorman, 2000). Moreover, SampEn has two advantages over ApEn, namely, data length independence and a relatively simple implementation. Similar to ApEn, SampEn is a measure of complexity (Richman and Moorman, 2000). The Shannon entropy provides a useful criterion for the analysis and comparison of probability distributions, which can act as a measure of the information of any distribution; namely, the wavelet entropy (WE) (Blanco et al., 1998). In this study, the total WE was defined as follows:

(3) $$\begin{array}{l} S_{WT}\;\equiv S_{WT}\left(p\right)=\underset{j<0}{\sum }p_{j}\cdot In\left[p_{j}\right] \end{array}$$

The WE can be used as a measure of the degree of order/disorder of the signal; thus, it can provide useful information on the underlying dynamical process associated with the signal.

### 2.4. ST-SBSSVM

**Figure 4:**
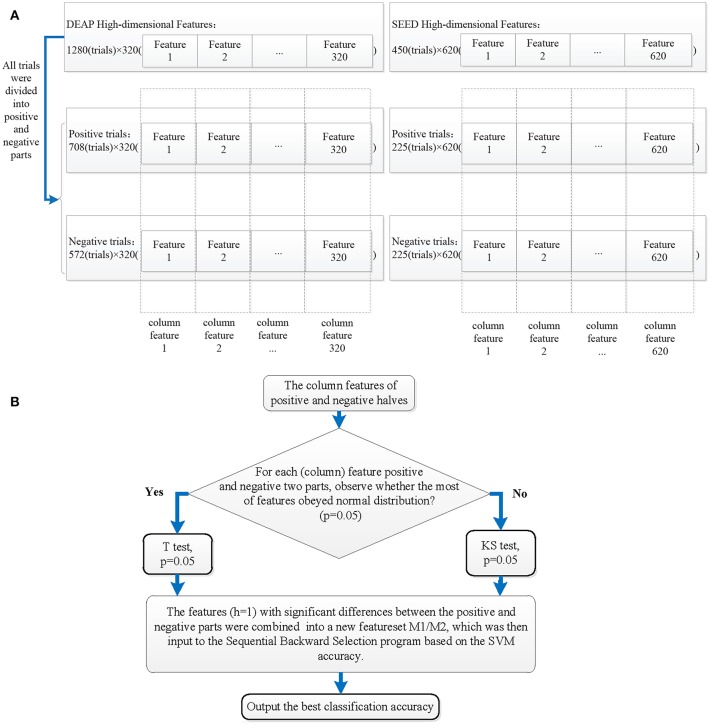
Processes of ST-SBSSVM method: **(A)** generation of column features of positive and negative halves along with the division of trials (rows), and **(B)** the ST-SBSSVM analysis of all column features.

The ST-SBSSVM method is a combination of the significance test, sequential backward selection, and support vector machine. In this study, the SVM based on the radial basis function (RBF) kernel was employed. The detailed fusion process is shown in Figure 4. Ten types of features from both public datasets were extracted, and high-dimensional features [*DEAP*, 1280(*trials, rows*) × 320(*features, cols*); and *SEED*, 450(*trials, rows*) × 620(*features, cols*)] were formed. If the sequential backward selection (SBS) method was directly employed for the analysis of the high-dimensional EEG features and SVM was used to determine the accuracy of the emotion classification of each feature combination, the computational overhead would be significantly large. Therefore, a method was developed to achieve a higher emotional recognition accuracy across the subjects than the SBS, namely the ST-SBSSVM. Moreover, the proposed method requires a significantly lower computational overhead for the analysis of high-dimensional EEG features. As shown in step 1 of Figure 4, each trial (row) of 1280(*trials, rows*) × 320(*features, cols*) and 450(*trials, rows*) × 620(*features, cols*) was in one-to-one correspondence with the positive and negative emotion labels. In step 2, according to the labels, all the trials (rows) were divided into two parts. The objective was to simultaneously divide each column feature into two parts. In step 3, the significance test was carried out from the first column feature to the final column feature for the column features that were divided into two positive and negative parts (the last column of the DEAP feature was the 320th column, and the last column of the SEED feature was the 620th column). It was then determined whether the majority of EEG column features, which were divided, were in accordance with the normal distribution. If the majority of EEGs were subject to the normal distribution, the student's *t*-test (*T*-test) was used for the divided column features; otherwise, the Kolmogorov–Smirnov (KS) test was used. The corresponding column features of the positive and negative significant difference (h = 1) were then selected. In step 4, after the significance test, the high-dimensional feature set was simplified, and the following was obtained:

(4) $$\begin{array}{l} M_{1}=1280\left(trials,rows\right)\times 68\left(features,cols\right) \end{array}$$

(5) $$\begin{array}{l} M_{2}=450\left(trials,rows\right)\times 227\left(features,cols\right) \end{array}$$

In step 5, M1 and M2 were inputted into the SVM-based SBS program. Sequential backward selection is a process that decreases the number of features, in which a feature is repeatedly eliminated until a final feature is remaining. In this manner, all the feature combinations were separately classified by the SVM. The data was normalized prior to the use of SVM modeling for emotion classification recognition, which helped to improve the convergence rate and accuracy of the model. In the SVM-based SBS program, a “leave-one-subject-out” verification strategy was employed. During each process, the data of one subject was considered as the test set, and the data of the other subjects were considered as the training set. The feature selection was carried out on the training set, and the performance was then evaluated on the test set. This procedure was iterated until the data of each subject had been tested. Moreover, this strategy can eliminate the risk of “overfitting”. In step 6, the average classification accuracy of the employed “leave-one-subject-out” verification strategy and SVM-based SBS program was outputted.

## 3. Results

**Figure 5:**
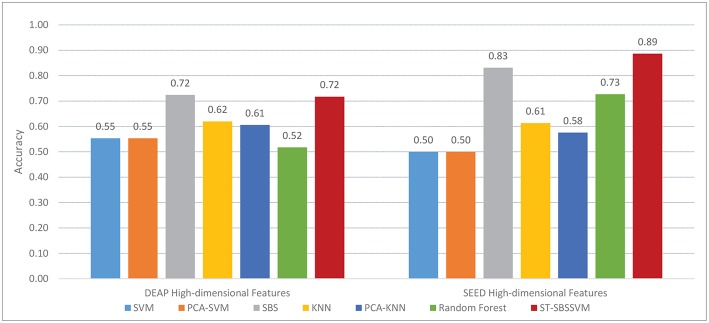
Accuracy results of valence classification using DEAP and SEED.

**Figure 6:**
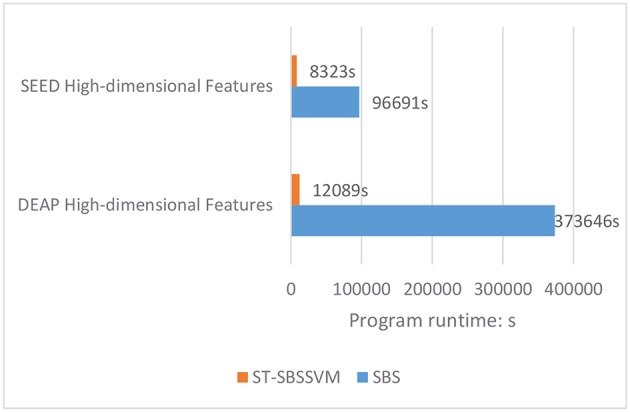
Comparison of runtime between ST-SBSSVM and SBS methods.

Figure 5 and Table 4 present a comparison between the valence classification recognition results of the ST-SBSSVM and those of common methods using the DEAP and SEED. For the consistency of the analysis of the two datasets, only cross-subject emotional recognition was carried out for the valence classification. The ST-SBSSVM method is an improvement of the SBS method; thus, the two methods were compared. Figure 5 and Table 4 present a comparison of the recognition accuracies of the two emotions. Therefore, Figure 6 presents a runtime comparison between the ST-SBSSVM and corresponding SBS program (using the corresponding method for emotion recognition on the same computer “DELL, intel(R) Core (TM) i5-4590 CPU @ 3.30 GHz, RAM-8.00 GB”). As shown in Figure 5 and Table 4, with respect to high-dimensional features, the accuracy of the ST-SBSSVM was improved by 12.4% (DEAP) and 26.5% (SEED) when compared with the common emotion recognition methods. From Table 4, it can be seen that with respect to high-dimensional features, the cross-subject emotion recognition accuracy of the ST-SBSSVM decreased by 0.42% (almost unchanged) using the DEAP, and it improved by 6% using the SEED. Figure 6 shows that the ST-SBSSVM decreased the program runtime by ~97 and 91% when compared with the SBS method.

**Table 4:** Comparison of Valence classification accuracy between ST-SBSSVM and common methods.

	**Difference from ST-SBSSVM accuracy (DEAP)**	**Difference from ST-SBSSVM accuracy (SEED)**
SVM	+17%	+39%
PCA-SVM	+17%	+39%
SBS	–0.42%	+6%
KNN	+10%	+28%
PCA-KNN	+11%	+31%
RF	+20%	+16%
The average difference		
from ST-SBSSVM accuracy	+12.4%	+26.5%

## 4. Discussions

**Figure 7:**
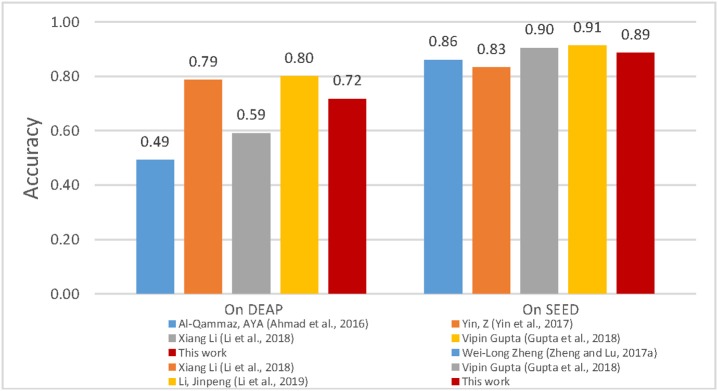
Comparison between valence classification accuracies of similar studies.

In this paper, a method that can effectively promote emotion recognition is proposed, namely, the ST-SBSSVM method. The proposed method was used to effectively analyze the high-dimensional EEG features extracted from the DEAP and SEED. The results of this study confirmed that the ST-SBSSVM method offers two advantages. First, the ST-SBSSVM can classify and identify emotions, with an improved emotion recognition accuracy. Because ST-SBSSVM performed the Significant Test by comparing the same column feature that had significant difference between positive and negative trials, a “leave-one-subject-out” verification strategy and SVM-based SBS program were then employed to carry out feature selection for those features with significant differences, and the best emotion classification accuracy was obtained. Second, the ST-SBSSVM and SBS methods exhibited similar emotion recognition results to those of the common emotion classification methods. Moreover, when using ST-SBSSVM and SBS to analyze high-dimensional features, ST-SBSSVM decreased the program runtime by ~90% when compared with SBS. The limitations of this study were as follows. The features extracted were relatively common, and these features were not the new features that significantly promoted emotion recognition in the most recent studies. In future work, the new features combined with ST- SBSSVM will be employed to investigate emotion recognition among subjects. In recent years, several EEG devices and data technologies were developed, such as using wearable EEG devices, for the collection of data in actual working environments (Jebelli et al., 2017b, 2018b; Chen et al., 2018). High quality brainwaves can then be extracted from the data collected by wearable EEG devices (Jebelli et al., 2017a). A stress recognition framework was proposed, which can effectively process and analyze EEG data collected from wearable EEG devices in real work environments (Jebelli et al., 2018c). These new developments comprise the scope of future research. Similar works are as follows. In (Ahmad et al., 2016), the empirical results revealed that the proposed genetic algorithm (GA) and least squares support vector machine (LS-SVM) (GA-LSSVM) increased the classification accuracy to 49.22% for valence using the DEAP. In Zheng and Lu (2017a), DBNs were trained using differential entropy features extracted from multichannel EEG data. As shown in Figure 7, the proposed method demonstrated a good performance and its accuracy was similar to those of achieved in similar studies with respect to the emotion classification recognition of the cross subjects using the same datasets. In summary, when compared with the most recent studies, this method developed in this study was found to be effective for emotional recognition across subjects.

## 5. Conclusions

For emotion recognition, a method is proposed in this paper that can significantly enhance the two-category emotion recognition effect; with a small computational overhead when using the corresponding program to analyze high-dimensional features. In this study, 10 types of EEG features were extracted to form high-dimensional features, and the proposed ST-SBSSVM method was employed, which can rapidly analyze high-dimensional features and effectively improve the accuracy of cross-subject emotion recognition, namely the ST-SBSSVM. The results of this work revealed that ST-SBSSVM demonstrates better accuracy with respect to emotion recognition than common classification methods. Compared with the SBS method, the ST-SBSSVM exhibited a higher accuracy of emotion recognition and significantly decreased the program runtime. In comparison to recent similar methods, the method proposed in this study is effective for emotional recognition across subjects. In summary, the proposed method can effectively promote the emotional recognition across subjects. This method can therefore contribute to the research of health therapy and intelligent human-computer interactions.

## Data Availability

The data set generated for this study can be provided to the corresponding author upon request.

## Author Contributions

FY developed the ST-SBSSVM method, performed all the data analysis, and wrote the manuscript. XZ, WJ, PG, and GL advised data analysis and edited the manuscript.

### Conflict of Interest Statement

The authors declare that the research was conducted in the absence of any commercial or financial relationships that could be construed as a potential conflict of interest.
